# Antibiotic heteroresistance in *Mycobacterium tuberculosis* isolates: a systematic review and meta-analysis

**DOI:** 10.1186/s12941-021-00478-z

**Published:** 2021-10-13

**Authors:** Mao Ye, Wen Yuan, Leila Molaeipour, Khalil Azizian, Alireza Ahmadi, Ebrahim Kouhsari

**Affiliations:** 1 Department of Pharmacy, Clinical Pharmaceutics Room, Sichuan Science City Hospital, Mianyang, 621000 China; 2 Sichuan College of Traditional Chinese Medicine, Mianyang, 621000 China; 3 Department of Epidemiology, School of Public Health, Iran University of Medical Sciences, Tehran, Iran; 4 Department of Clinical Microbiology, Sirjan School of Medical Sciences, P.O. Box 78169-16338, Sirjan, Iran; 5 Laboratory Sciences Research Center, Golestan University of Medical Sciences, Gorgan, Iran; 6 Department of Laboratory Sciences, Faculty of Paramedicine, Golestan University of Medical Sciences, Gorgan, Iran; 7 Laboratory Sciences Research Center, Faculty of Paramedical Sciences, Golestan University of Medical Sciences, Negative Floor 1, Gorgan-Sari Road, P.O. Box: 4918936316, Gorgan, Golestan Province Iran

**Keywords:** Drug resistance, Heteroresistance, Isoniazid, Rifampin, Fluoroquinolones

## Abstract

**Background:**

*Mycobacterium tuberculosis* (MTB) is responsible for tuberculosis; that continues to be a public health threat across the globe. Furthermore, increasing heteroresistance (HR)-the presence of resistant and susceptible isolates among MTB strains- has been reported from around the world. This phenomenon can lead to full resistance development and treatment failure.

**Methods:**

We systematically searched the relevant studies in PubMed, Scopus, and Embase (Until October 21, 2020). The study outcomes revealed the weighted pooled prevalence of antibiotic HR in MTB isolates with subgroup analysis by year, quality of study, and heteroresistance detection method.

**Results:**

A total of 38 studies which had investigated MTB isolates were included in the meta-analysis. Geographically, the highest number of studies were reported from Asia (n  =  24), followed by Africa (n  =  5). Nineteen studies reported HR to isoniazid, with a weighted pooled prevalence of 5% (95% CI 0–12) among 11,761 MTB isolates. Also, there is no important trend for the subgroup analysis by the study period (2001–2014 vs 2015–2017 vs 2018–2020). HR to rifampin was reported in 17 studies, with a weighted pooled prevalence of 7% (95% CI 2–14) among 3782 MTB isolates. HR to fluoroquinolone and ethambutol were reported in 12 and 4 studies, respectively, with weighted pooled prevalence of 10% and 1% among 2153 and 1509 MTB isolates, correspondingly.

**Conclusion:**

Based on our analysis, HR in MTB isolates with different frequency rate is present worldwide. Thus, the selection of appropriate and reliable methods for HR detection is crucial for TB eradication.

**Supplementary Information:**

The online version contains supplementary material available at 10.1186/s12941-021-00478-z.

## Introduction

*Mycobacterium tuberculosis* (MTB) is responsible for tuberculosis (TB), one of the oldest recognized infections and top 10 causes of death among infectious agents in mankind. Every year, 10 million people are infected with this bacterium worldwide [1, 2]. Between 2018 and 2020, 40 million new cases of TB were detected [3]. Globally, more than 1.2 million deaths related to TB infection occurred in HIV-negative (1.1–1.3 million) and HIV-positive (2,08,000) people during 2019 [3]. Therefore, TB still is, as it has always been, a serious threat to public health.

Isoniazid (INH), rifampin (RIF), fluoroquinolones (FQs), and ethambutol (EMB) are the most effective drugs/drug classes in the standard TB treatment protocol. Not only, the emergence of drug resistance has become an alarming global problem [4] imposing a significant impact on the circulation of *M. tuberculosis *across the world, but also the resistant isolates are considered the main barrier to TB control and its eradication. Every year, nearly half a million people developed rifampin-resistance and multidrug-resistant TB (MDR-TB; resistant to at least INH and RIF) [5]. Extensively drug-resistant MTB (XDR-TB) is more menacing than MDR; XDR-TB strains are defined as resistant to INH, RIF, FQs, and one aminoglycoside injectable agent. In 2018, 14,000 XDR-TB strains were isolated worldwide [6]. However, the prevalence of drug-resistant TB is unknown [7].

In many cases, detection of MTB drug-resistant isolates in clinical samples is difficult since drug-susceptible and drug-resistant isolates coexist [8, 9], which may result in masking drug-resistant isolates by drug-susceptible ones. This phenomenon referred to as heteroresistance (HR) [10] is common in MTB and considered one of the major steps in the development of drug-resistance in bacterial isolates [11]. HR may arise from a mixed infection, when resistant and susceptible strains infect a person at the same time, or while single clone changes from a susceptible strain to resistant by undergoing genetic mutation under antibiotic pressure [12].

In term of clonality, both of these phenomena are classified as polyclonal HR. Although the susceptible and resistant strains are available in polyclonal HR but resistant strains are not seen when pure clones are analyzed by conventional methods. On the other hand, origin of heterogeneity could be a single clone that has both susceptible and resistant populations and considered monoclonal HR. In contrast to previous cases, in monoclonal HR, the HR phenotype is detectable when pure clones are analyzed [12]. Some studies rejected correlation between HR and treatment failure [13, 14], nevertheless several other studies and increasing evidence linked HR and treatment failure in different bacteria [15, 16]. Moreover, HR has been described for several antibiotics and it is responsible for limited treatment options and also increasing rates of treatment failure in TB patients [17]. Since, HR frequency rates of MTB isolates are not very well documented, the main aim of this review and meta-analysis is to bring a comprehensive data analysis on the prevalence of heteroresistant MTB against commonly used antimicrobial agents together, including INH, RIF, FQs, and EMB. To the best of our knowledge, our meta-analysis describes the first cross study report on the prevalence of HR in MTB isolates.

## Methods

### Guidelines

This review is reported accordant with the preferred reporting items for systematic reviews and meta analyses guidelines (PRISMA) [18].

### Search strategy and study selection

MEDLINE (PubMed), Scopus, and Embase were searched for relevant articles (Until 21, October 2020) by using the following keywords: (“*Mycobacterium tuberculosis*” or “*M. tuberculosis*’’ or “MTB” or “tuberculosis” or “TB”) AND (“heteroresistance” or “hetero-resistance” or “heteroresistant” or “hetero-resistant”) in the Title/Abstract/Keywords fields. No limitation was used while searching databases, but inclusion of the study in our full analysis required at least the abstract to be available in English. The records found through database searching were merged and the duplicates were removed using EndNote X7 (Thomson Reuters, New York, NY, USA).

### Selection criteria and data extraction

Three reviewers (AKH, LM, EK) screened all titles and abstracts independently and excluded irrelevant data, then they independently assessed the remaining articles for inclusion. Discrepancies were resolved by discussion and a fourth author (EK) acted as arbiter. The information extracted from each study included was (1) author, (2) publication year, (3) type of samples (pulmonary or extrapulmonary), (4) number of isolates, (5) the method of heteroresistance detection [line-probe assay (LPA), sequencing-based methods, and other], and (6) heteroresistance rates (Additional file 2). Studies were excluded if they met the following conditions: (1) HR were not clearly reported; or (2) data on HR were from a meta-analysis and/or systematic review, non-original research, or conference abstract.

### Quality assessment

The quality of the included studies was assessed by 2 reviewers (AKH, EK) independently, using an adapted version of the tool proposed by the Newcastle–Ottawa assessment scale for cross-sectional studies [19]. A score ranging from 0 to 7 points was attributed to each study (7 points: high quality,  ≤  6 points: low quality). A third reviewer (LM) acted as an arbiter adjudicated in any cases where there was disagreement.

### Definitions

HR refers to the occurrence of the populations of both drug-susceptible and drug-resistant isolates within the same clinical sample [20, 21].

### Statistical analysis

To analyse and combine the results of different studies, in each study, the prevalence of HR was considered a binomial distribution and its standard error was calculated by this distribution. Heterogeneity of studies was assessed using Cochran’s Q test and I^2^ index. Due to the heterogeneity of the studies, a random-effects model was used in the meta-analysis. Sensitivity analysis was used to investigate the heterogeneity sources between studies. Analysis was performed using Stata/SE software, v.14 (StataCorp, College Station, TX). All statistical interpretations were reported on a 95% confidence interval (CI) basis.

### Study outcome

The main outcome of the study was the weighted pooled prevalence of HR to INH, RIF, FQs, and EMB in MTB isolates. A subgroup analysis was performed as; (1) HR method, (2) year of publication (2001–2014, 2015–2017, and 2018–2020), and (3) the quality of the studies (high quality and low quality).

## Results

### Results of the systematic literature search

**Fig. 1:**
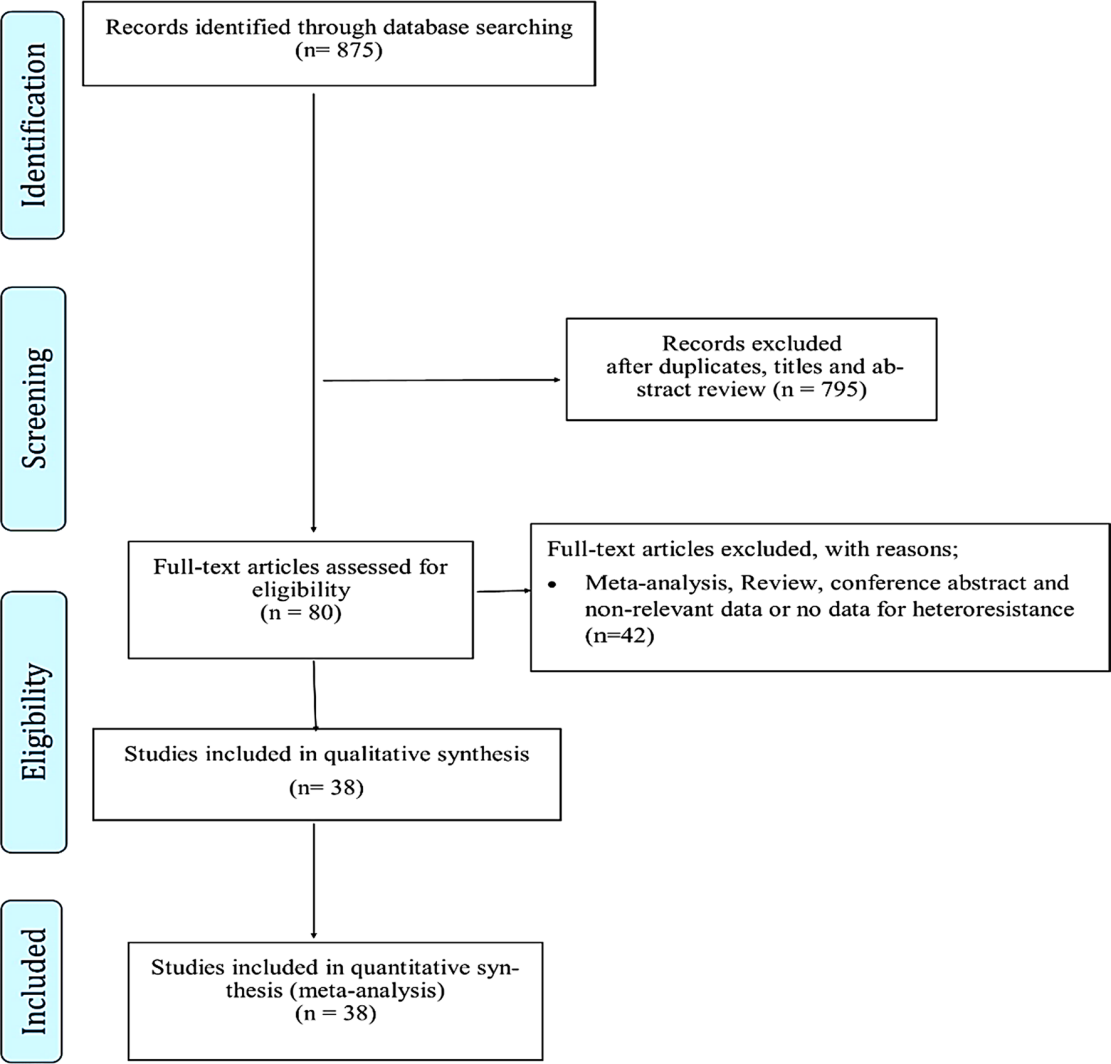
Flow chart of study selection

Thirty-eight studies [1, 4, 11, 22–56] comprising 19,205 MTB isolates were included in this systematic review and meta-analysis (Fig. 1, Additional file 2). All 38 studies had a cross-sectional design. Geographically, the HR rates were reported from Asia (n  =  24), Africa (n  =  5), and Europe (n  =  3), (see Additional file 2). The studies included in this meta-analysis evaluated HR to isoniazid, rifampin, fluoroquinolones, and ethambutol.

### Meta-analysis results

#### Isoniazid

HR to INH was reported in 19 studies, with a weighted pooled prevalence of 5% (95% CI 0–12) among 11,761 MTB isolates; a substantial heterogeneity was I^2^  =  99.42% (Table 1). Sensitivity analysis was performed based on the quality of the articles, HR assay, and the year of the study. To analyze the trends for changes in the prevalence of HR to INH in more recent years, we performed a subgroup analysis for three periods (2001–2014, 2015–2017, and 2018–2020) (Table 1, Additional file 1). However, there is no important trend for the subgroup analysis by the mentioned study period (2001–2014 vs 2015–2017 vs 2018–2020). LPA (n = 16) and sequencing-based methods (n  =  2) were the most frequent HR methods. The prevalence of HR to INH was 5% (95% CI 1–15) using LPA, 0% (95% CI 0–1) using sequencing-based methods, and 1% (95% CI 0–6) using other-methods (e.g., RT-PCR) (Table 1).

**Table 1:** Prevalence of antibiotic heteroresistance in *M. tuberculosis* isolates based on quality, publication year, and heteroresistant assay

Antibiotic	Category	Subcategory	No. of studies	No. of heteroresistant isolates	No. of heteroresistant	Prevalence (%) (95% CI)	I^2^
Isoniazid	Overall		19	11,761	1236	5 (0–12)	99.42
	Heteroresistance assay	Line-probe assay (LPA)	16	10,266	1219	5 (0–15)	99.49
		Sequencing-based methods	2	1400	16	0 (0–1)	–
		Other	1	95	1	1 (0–6)	–
	Year	2001–2014	6	620	56	9 (1–23)	95.06
		2015–2017	8	6035	43	2 (0–5)	93.29
		2018–2020	5	5106	1137	4 (0–27)	99.71
	Quality	High	7	4220	1175	9(0–27)	99.02
		Low	12	7541	61	2 (1–5)	92.68
Rifampin	Overall		17	3782	600	7 (2–14)	97.59
	Heteroresistance assay	Line-probe assay (LPA)	9	2511	526	9 (2–20)	97.43
		Sequencing-based methods	6	1001	71	6 (0–16)	96.02
		Other	2	270	3	1 (0–2)	–
	Year	2001–2014	6	808	102	12 (3–26)	95.84
		2015–2017	7	1269	28	2 (1–4)	65.52
		2018–2020	4	1705	470	10 (1–28)	93.42
	Quality	High	7	2479	506	9 (1–23)	98.49
		Low	10	1303	94	6 (1–12)	93.13
Fluoroquinolones	Overall		12	2153	113	10 (3–19)	96.20
	Heteroresistance assay	Line-probe assay (LPA)	4	350	25	9 (0–24)	92.26
		Sequencing-based methods	7	1711	80	10 (1–27)	97.40
		Other	1	92	8	9 (4–16)	–
	Year	2001–2014	5	636	81	12 (3–26)	94.48
		2015–2017	4	181	22	11 (1–29)	86.59
		2018–2020	3	1336	10	5 (0–22)	–
	Quality	High	6	401	38	10 (5–18)	74.92
		Low	6	1752	75	9 (0–25)	97.79
Ethambutol	Overall		4	1509	9	1 (0–4)	78.23

*I*^*2*^ the percentage of variance in a meta-analysis that shows study heterogeneity

#### Rifampin

HR to RIF was reported in 17 studies, with a weighted pooled prevalence of 7% (95% CI 2–14; I^2^  =  97.59%) among 3782 MTB isolates (Table 1). Sensitivity analysis was performed based on the quality of the articles, HR assay, and the year of the study. The prevalence of HR to RIF was found to be 12% (95% CI 3–26) during 2001–2014, dropping to 2% (95% CI 1–4) in 2015–2017, but growing back to 10% in 2018–2020 (95% CI 1–28) (Table 1; Additional file 1). LPA (n  =  9) and sequencing-based methods (n  =  6) were the most frequent HR methods. The prevalence of HR to RIF using LPA was 9% (95% CI 2–20), 6% (95% CI 0–16) using sequencing-based methods, and 1% (95% CI 0–2) using other-methods (Table 1).

#### Fluoroquinolones

HR to FQs was reported in 12 studies, with a weighted pooled prevalence of 10% (CI 95% 3–19; I^2^  =  96.2% (among 2153 MTB isolates (Table 1). Sensitivity analysis was performed based on the quality of the articles, HR assay, and the year of the study. The results of sensitivity analysis based on the quality of articles showed complete homogeneity between the results in high quality studies (I^2^  =  74.92%), and substantial heterogeneity in low quality articles (I^2^  =  97.79%) (Table 1, Additional file 1). The prevalence of HR to FQs was found to be 12% (95% CI 3–26) during 2001–2014, reaching to 11% (95% CI 1–29) in 2015–2017, but dropping at 5% in 2018–2020 (95% CI 0–22) (Table 1; Additional file 1).

LPA (n  =  4) and sequencing-based methods (n  =  7) were the most frequent HR methods. The prevalence of HR to FQs using LPA was 9% (95% CI 0–24), 10% (95% CI 1–27) using sequencing-based methods, and 9% (95% CI 4–16) using other-methods (Table 1).

#### Ethambutol

**Fig. 2:**
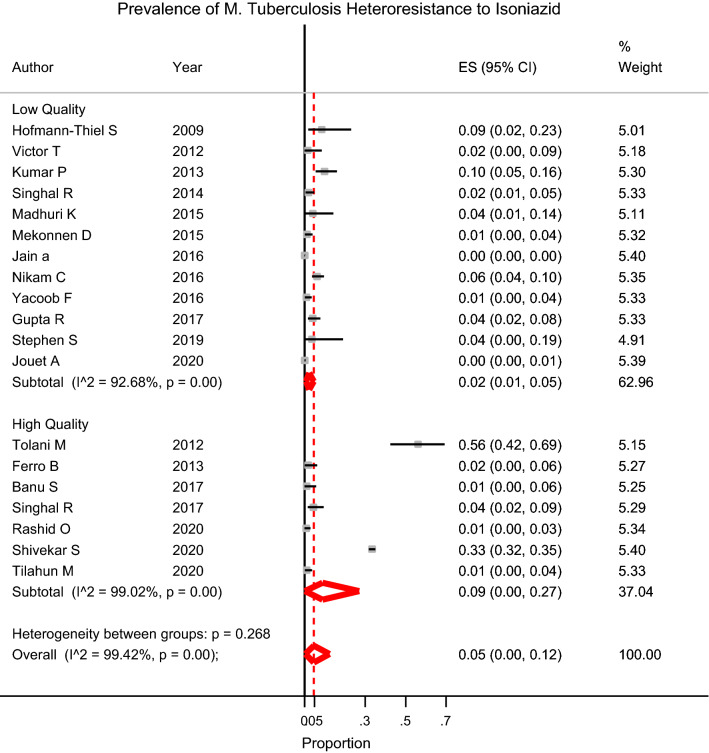
Forest plot of heteroresistance rate to isoniazid based on quality

HR to EMB was reported in 4 studies, with a weighted pooled prevalence of 1% (95% CI  =  0.01–0.04) among 1509 MTB isolates; a substantial heterogeneity was I^2^  =  78.23% (Fig. 2).

## Discussion

The prevalence of HR to INH, RIF, FQs, and EMB was the main outcome of this meta-analysis. INH, RIF, FQs, and EMB are included in the first and second line of TB therapy protocols. Development of monoresistance, MDR and specific HR in clinical isolates has been reported in previous studies [1, 57]. HR is an initial step to change from susceptible to monoresistant and/or MDR [58]. All different resistant forms are responsible for treatment failure, thus a more comprehensive understanding of the emergence, spread, and methods for detection of HR is critical. Here HR is defined as the presence of susceptible and resistant strains in one sample, and can be detected by conventional phenotypic and genotypic drug susceptibility test—DST-[Lowenstein–Jensen (LJ)]. As expected, genotypic DST methods provide results with more sensitivity and reliability [58]. As previously defined, no standard methods for the detection of heteroresistant strains exist, therefore, different methods are applied for HR detection including phonotypical methods (Etest, the disc diffusion, and the population analysis profile (PAP) test, and molecular methods (LPA, sequencing, MTBDRplus, and iPLEX Gold assay). Etest and disc diffusion tests reflect poor specificity and sensitivity. The PAP test is reliable method but difficult to perform, due to high cost and labor intensity [12]. In contrast, molecular test is easier to perform. Nonetheless, Folkvardsen et al. [9] demonstrated that various methods have different sensitivity and *Mycobacterium* growth indicator tubes (MGIT) DST is the most sensitive method in detection of MTBDR and heteroresistant MTB. In all articles included in our study, molecular methods were applied for detection of HR (such, as LPA, sequencing, MTBDRplus, iPLEX Gold assay, and etc.) (Additional file 2). Analyzing the efficiency of different detection methods is so complicated and expensive. Therefore, the clear data on their efficiency is limited [9]. HR prevalence to RIF (7%) did not differ significantly compared to INH (5%). The prevalence of HR is varied among different studies. For example, the low prevalence of HR to INH and RIF (less than 1%) was reported in some studies [25, 59, 60], while discordant results were shown by other studies (higher than 5%) [12, 61, 62]. On the other side, a very high prevalence of HR to INH and RIF (20%) in 35 clinical samples has been reported by Hofmann-Thiel et al. [57]. Same results were demonstrated by other studies [63, 64]. The most significant factors related to the differences in HR prevalence may be variety in sample size and detection methods. Based on subgroup analysis in the current study, no rising or gradually decreasing of HR prevalence was observed in the last 20 years. Probably this is associated with improvements in the detection methods for HR isolates resulting in appropriate therapy protocols and satisfactory outcomes. FQs are bactericidal drugs against MTB, and known as a major member of therapy protocols against MDR and XDR isolates [23, 65]. But recently, the high prevalence of FQs-resistant TB has been detected which leads to treatment failure [66, 67]. Results of our meta-analysis explain that the prevalence of HR to FQs (10%) is higher in comparison to other investigated drugs, including INH, RIF, and EMB. Also, a high proportion of (20–38%) HR to FQs has been observed in previous studies [54, 68–70]. However, discordant results were obtained by other researchers and a low proportion (about 1%) of HR to FQs has also been reported [4, 71, 72]. Because of a very high proportion of HR or the risk of bias, we eliminated five articles in different subgroup analysis [54, 63, 64, 73, 74]. In fact, several factors are involved in this discrepancy in the proportion of HR among published studies. Firstly, study population and the origin of isolates (direct specimen or culture) can influence the HR diagnosis [75]. Secondly, only MDR/XDR isolates or patients with treatment failure and relapses were included in some studies [65, 70]. Thirdly, as previously mentioned, different methods possess varied sensitivity, thus the applied method is a major factor affecting the detection of HR [57]. Finally, geographic study area could be another factor, for example HR prevalence in particular regions like Uzbekistan and India is higher, because overall TB prevalence is higher in these regions [57, 75].

## Conclusion

In summary, results obtained by this meta-analysis have provided a comprehensive insight on the proportion of HR to the main drugs employed against MTB isolates. Based on our analysis, HR in MTB isolates with different frequency rate is present worldwide. On the other hand, HR is the main stage in the development of fully resistant isolates in TB patients. Therefore, to tackle this problem, the control of emerging resistance and also, decreasing antibiotics resistance rate, via fast and appropriate detection methods for HR diagnosis is crucial. Reliable HR detection method is an urgent need for selection of the correct therapy protocol and TB eradication.

## Supplementary Information

**Additional file 1.** Characteristics of included studies.

**Additional file 2.** Forest plots of antibiotic heteroresistance rate in *M. tuberculosis* isolates based on quality, publication year, and heteroresistant assay.

## Data Availability

All the data in this review are included in the manuscript.

## Abbreviations

MTB: *Mycobacterium tuberculosis*
HR: Heteroresistance
TB: Tuberculosis
INH: Isoniazid
RIF: Rifampin
FQs: Fluoroquinolones
EMB: Ethambutol
XDR-TB: Extensively drug-resistant MTB
PRISMA: Preferred reporting items for systematic reviews and meta analyses guidelines
LPA: Line-probe assay
CI: Confidence interval
PAP: Population analysis profile
MDR-TB: Multidrug-resistant TB
