# Supplementary material for: Antibiotic heteroresistance in Mycobacterium tuberculosis isolates: a systematic review and meta-analysis
Source: Ann Clin Microbiol Antimicrob. 2021 Oct 13;20:73. doi: 10.1186/s12941-021-00478-z (PMC8513340; doi:10.1186/s12941-021-00478-z)
Supplement: Supplementary file 1 — Additional file 1. Characteristics of included studies. [file 12941_2021_478_MOESM1_ESM.docx]

**Antibiotic Heteroresistance in *Mycobacterium tuberculosis*: A Systematic Review and Meta-Analysis**

**Isoniazid**

**Rifampin**

**Fluoroquinolones**

**Ethambutol**
